# Supplementary material for: DNA2 and MSH2 cooperatively repair stabilized G4 and allow efficient telomere replication
Source: Nat Commun. 2025 Sep 26;16:8519. doi: 10.1038/s41467-025-63505-z (PMC12474859; doi:10.1038/s41467-025-63505-z)
Supplement: Supplementary file 1 — Supplementary Information [file 41467_2025_63505_MOESM1_ESM.pdf]

Supplementary Table 1: List of top ECCs that dock to the G4 structure

CAS NO.	Common Name	Category	Docking Score (kcal/mol)
11056-06-7	Bleomycin	FDA drug	-14.871
213416-70-7	PIPER*	Small mol.	-10.544
24106-89-2	Pigment Red 123*	CDR comp.	-9.145
67786-25-8	Stilbenedisulfonate	CDR comp.	-7.326
1162-65-8	Aflatoxin B1	Carcinogen	-6.365
112484-44-3	Polyaza dye	CDR comp.	-6.014
553-12-8	Protoporphyrin Ⅸ	Metabolite	-5.076
11003-38-6	Capreomycin	FDA drug	-5.026
146939-27-7	Ziprasidone	FDA drug	-4.476

*Perylene derivatives

Supplementary Table 2: List of oligonucleotides for DNA2 nuclease assay and Polδ extension assay

oligonucleotide name	sequence (5' to 3')
G4B/FAM-G4B	GTTAAGATAGGTCTGCTTGGCATGTCAATTAGG GTTAGGGTTAGGGTTAGGGCTCTGTGGTTGAG GCAGAGTCCTTAAGC
G4B-T	GCTTAAGGACTCTGCCTCAACCACAGAGCCCT AACCCTAACCCTAACCCTAATTGACATGCCAAG CAGACCTATCTTAAC
Random/FAM-random	GTTAAGATAGGTCTGCTTGGCATGTCAAGGTTT CTAAAGAAGCCGACGGTAGCTCTGTGGTTGAG GCAGAGTCCTTAAG
FAM-G4B-primer	GCTTAAGGACTCTGCC
G4B (blocking 3'-end cleavage)	TGCTCGTTTTGTTTGGTCTGCTTGGCATGTCAA TTAGGGTTAGGGTTAGGGTTAGGGCTCTGTGG TTGAGGCAGAGTCCTTAAGC
Complemented-G4B (blocking 3'-end cleavage)	TGCTCGCTTTTGGTCTCTGCCTCAACCACAGAG CCCTAACCCTAACCCTAACCCTAATTGACATGC CAAGCAGACCTATCTTAAC

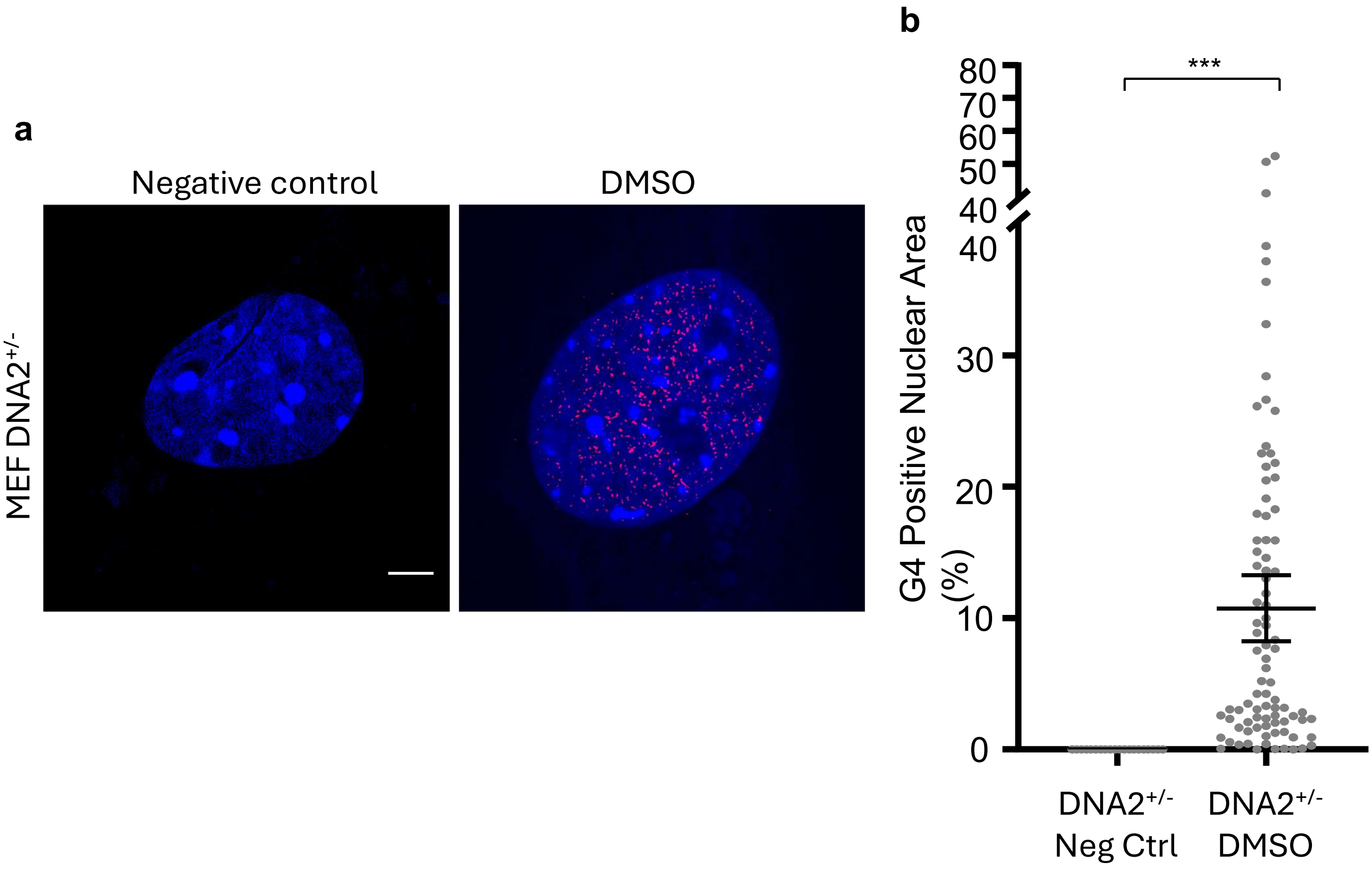

Supplementary Fig. 1 | The specificity of G4 antibody to recognize G4 in cells. a Representative images for G4 staining with negative control (G4 substrate neutralized G4 antibody) or G4 specific antibody in MEF DNA2$^{+/-}$ cells treated with DMSO. Scale bar = 5 µm. Quantification of positive G4 foci are presented as the percentage of G4 positive per nuclear area (n=20, 89 cells). P-value was calculated by two-tailed Student's t-test (p=0.0001).

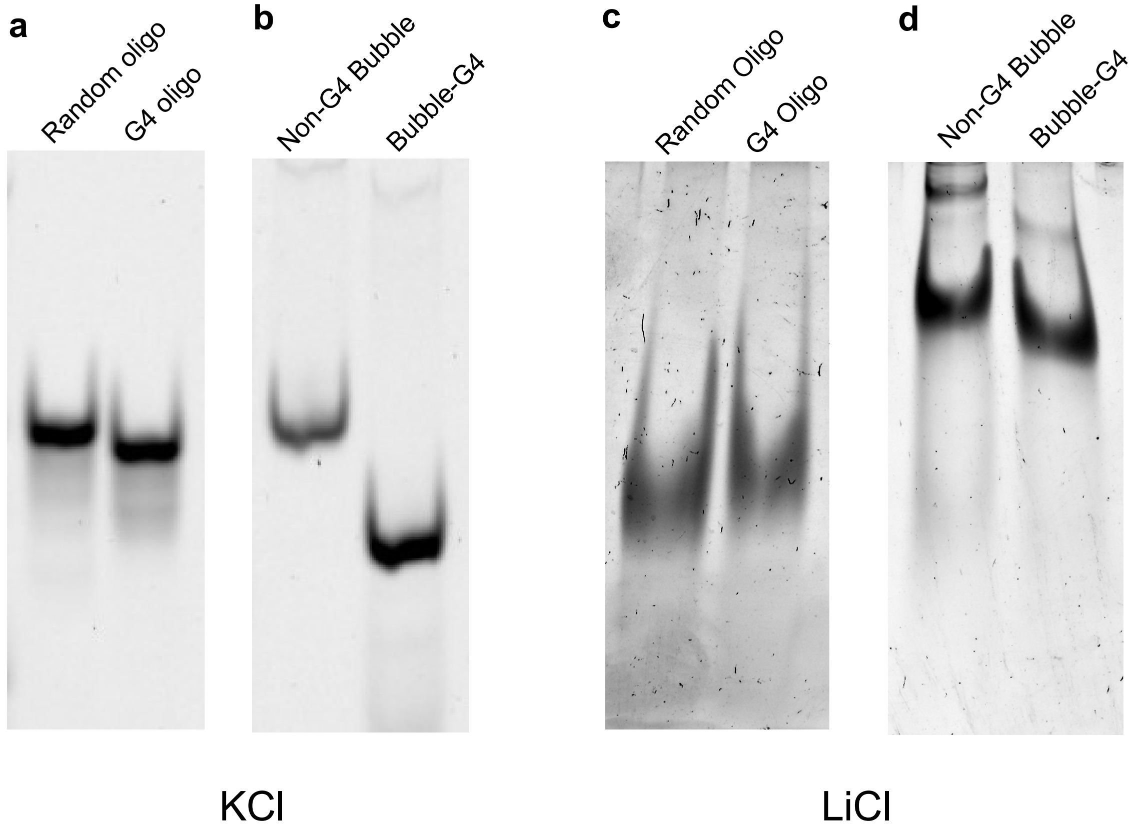

Supplementary Fig. 2 | Native PAGE confirms the formation of G4 DNA substrates. a, b. Single-stranded (a) and double-stranded (b) DNA substrates were annealed using an oligo of random DNA sequence or an oligo containing a G4-forming sequence in KCl. The formation of G4 was analyzed using 8% native PAGE. **c, d.** The same assay was performed with LiCl and shows little separation between random and G4-forming sequence DNA, indicative of G4 instability in the presence of lithium instead of potassium.

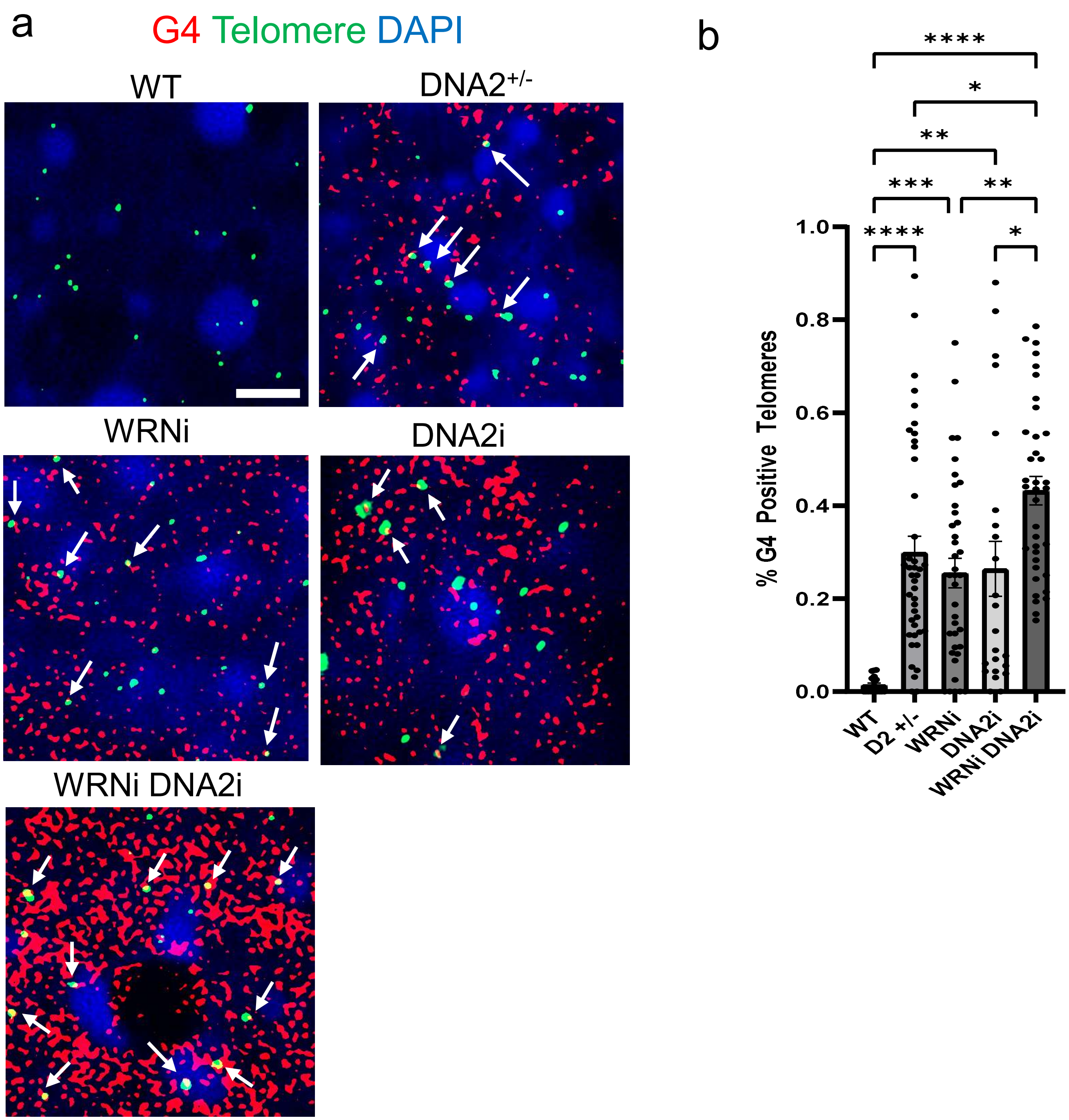

Supplementary Fig. 3 | Telomere G4 staining reveals helicase and nuclease dependent G4 resolution. a. WT and DNA2 +/- MEF cells were treated either with DMSO or WRN or DNA2 inhibitors. Arrows point to co-localization of G4 and telomere (Scale = 2 µm)**. b.** The fraction of G4 positive telomeres was assessed across multiple conditions (n=18, 42, 38, 23, 37 cells). P-value was calculated using one-way ANOVA (WT vs. DNA2+/- $p<0.0001$; WT vs. WRNi $p=0.0005$; WT vs. DNA2i $p=0.0012$; WT vs. WRNi DNA2i $p<0.0001$; DNA2+/- vs.WRNi DNA2i $p=0.0336$; WRNi vs. WRNi DNA2i $p=0.0019$; DNA2i vs. WRNi DNA2i $p=0.0174$). DNA2 heterozygosity, along with separate DNA2 and WRN inhibition all lead to a significant increase in G4 positive telomeres, though the combination of both inhibitors lead to an even greater increase, showing that the DNA2 and helicase pathways of G4 resolution are separate.

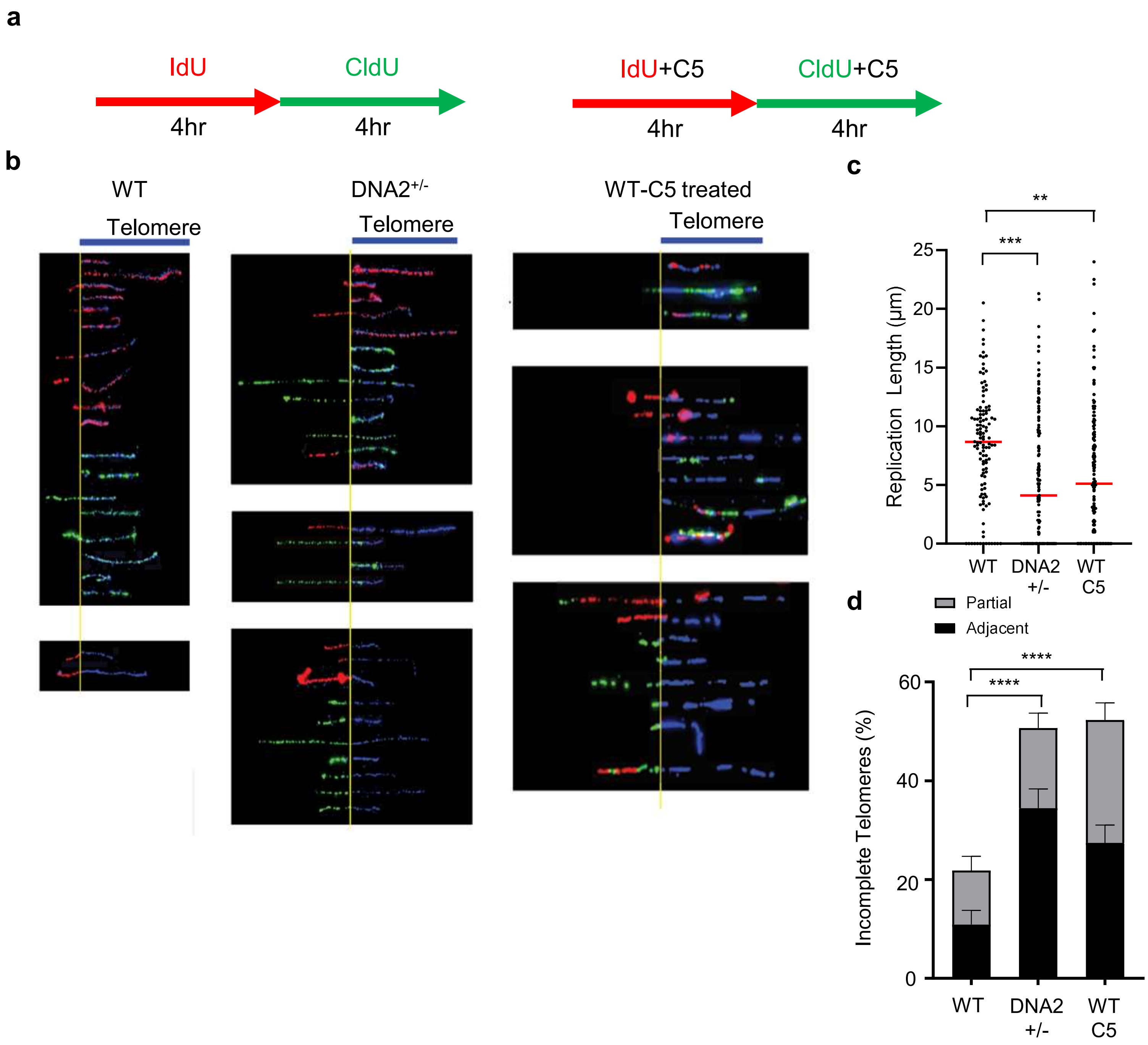

Supplementary Fig. 4 | SMARD assays on MEF cells of WT, DNA2$^{+/-}$ and WT treated with C5. a: Scheme of the IdU (red) and CldU (green) pulse labeling and C5 treatment; **b**: Representative images for SMARD assay results. WT and DNA2$^{+/-}$ MEF cells or WT MEF cells treated with C5 were labeled with IdU/CldU. DNA was digested with a cutting enzyme and isolated. Telomeric DNA was identified by a TelC-Biotin probe and fluorescently labeled Avidin (blue). Replicating DNA, which was incorporated with IdU (red) and/or CldU (green), was detected using anti-BrdU antibodies and DyLight-488 or Alexa-568 conjugated secondary antibodies; **c:** The length of replicated tracks in telomeres (n=118, 148, 153 fibers). P-value was calculated using two-tailed Student's t-test (WT vs. DNA2+/- p=0.0008, WT vs. WT C5 p=0.0035); **d:** Fractions of all measured telomeres (n=119, 148, 153 fibers) showing the fraction of telomeres with incomplete replication, both partial replication and replication stalling immediately adjacent to telomeres. P-value was calculated using two-sided chi-square test (p<0.0001)

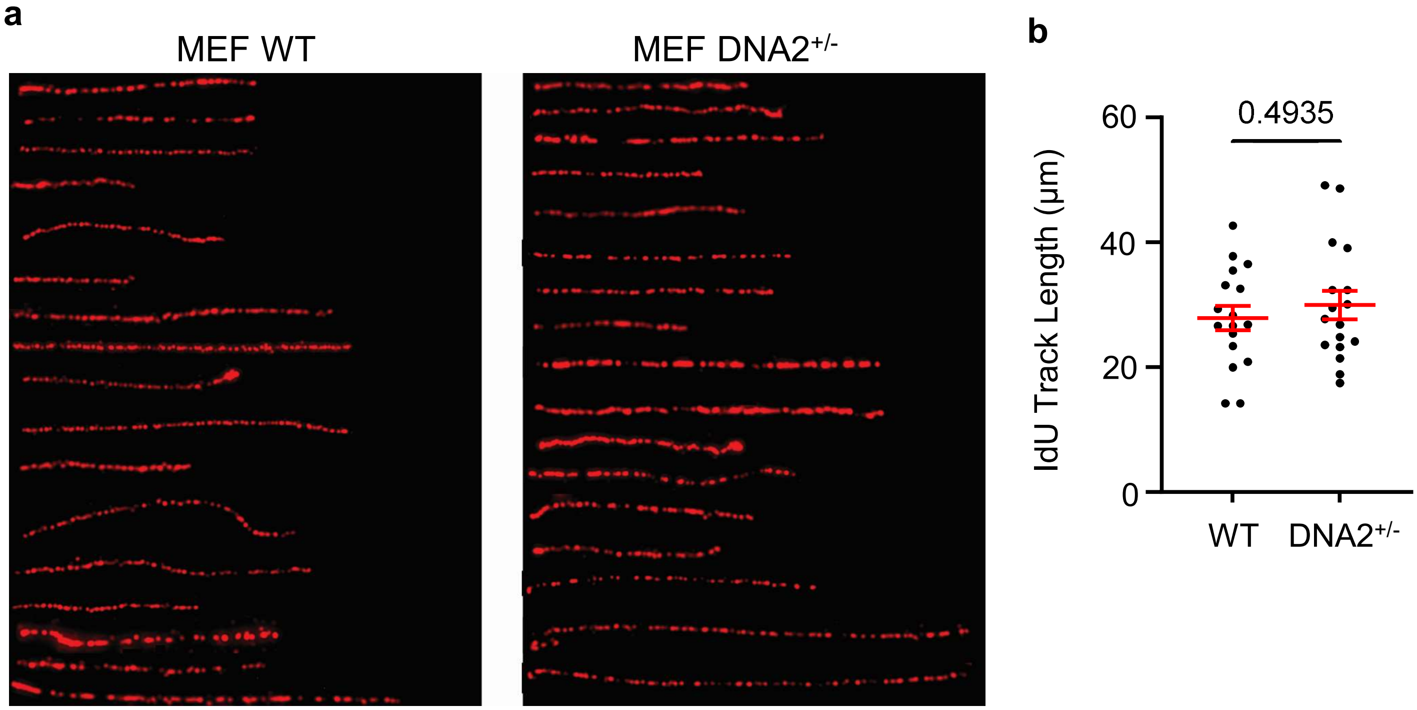

Supplementary Figure 5. DNA2 has a limited role in replication rates outside of DTR regions. **a.** SMARD Fibers that do not contain telomeres were compared in both WT and DNA2$^{+/-}$ MEF; b. No significant difference in replication rates were observed, consistent with previous findings that DNA2's role in replication of nuclear DNA is limited to difficult to replicate regions, with a limited role in most nuclear loci (n=17, 17 fibers). P-value was calculated using two-tailed Welch's t-test.

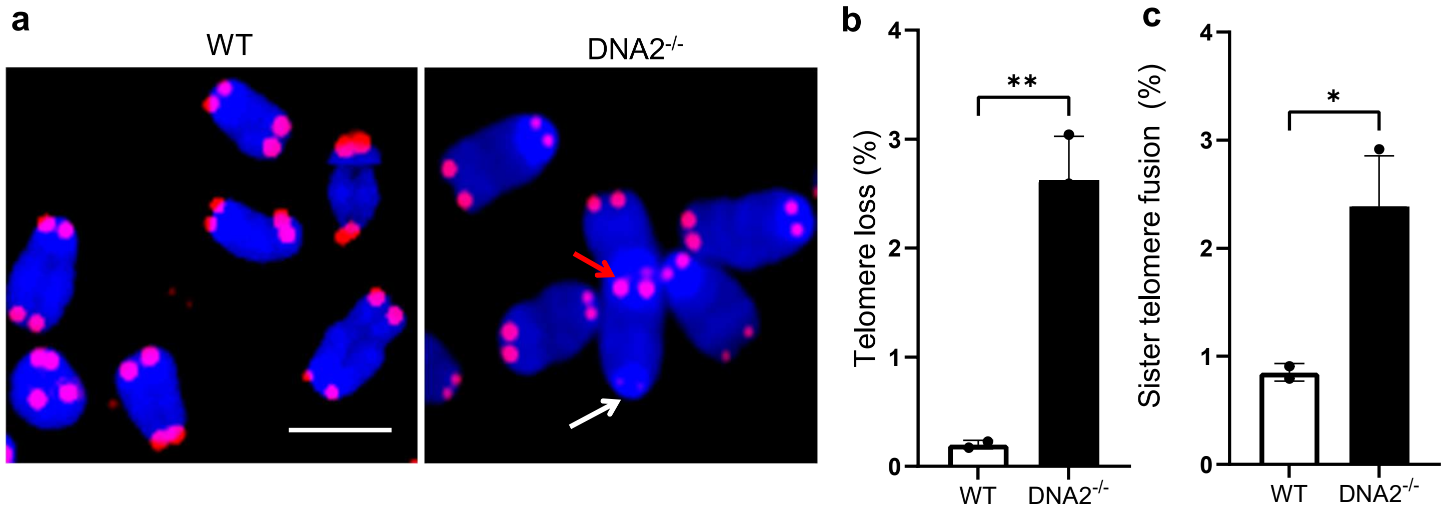

Supplementary Fig. 6 | Telomere stability in WT and DNA2$^{-/-}$ mouse ES cells. a Representative telomere FISH images showing telomeres in WT and DNA2$^{-/-}$ mouse ES cells. DNA was counter-stained by DAPI (blue). Telomere loss (signal-free ends) or sister telomere fusion are indicated by white and red arrows, respectively. Scale bar = 5 μm; **b, c** Quantification of telomere loss and sister telomere fusion in mouse ES cells. Values are mean ± st.d from WT ES cells (n=1980 sister chromatid pairs) and DNA2$^{-/-}$ ES clones (n=3420 sister chromatid pairs). * $p < 0.05$, ** $p < 0.01$. Student's t test.

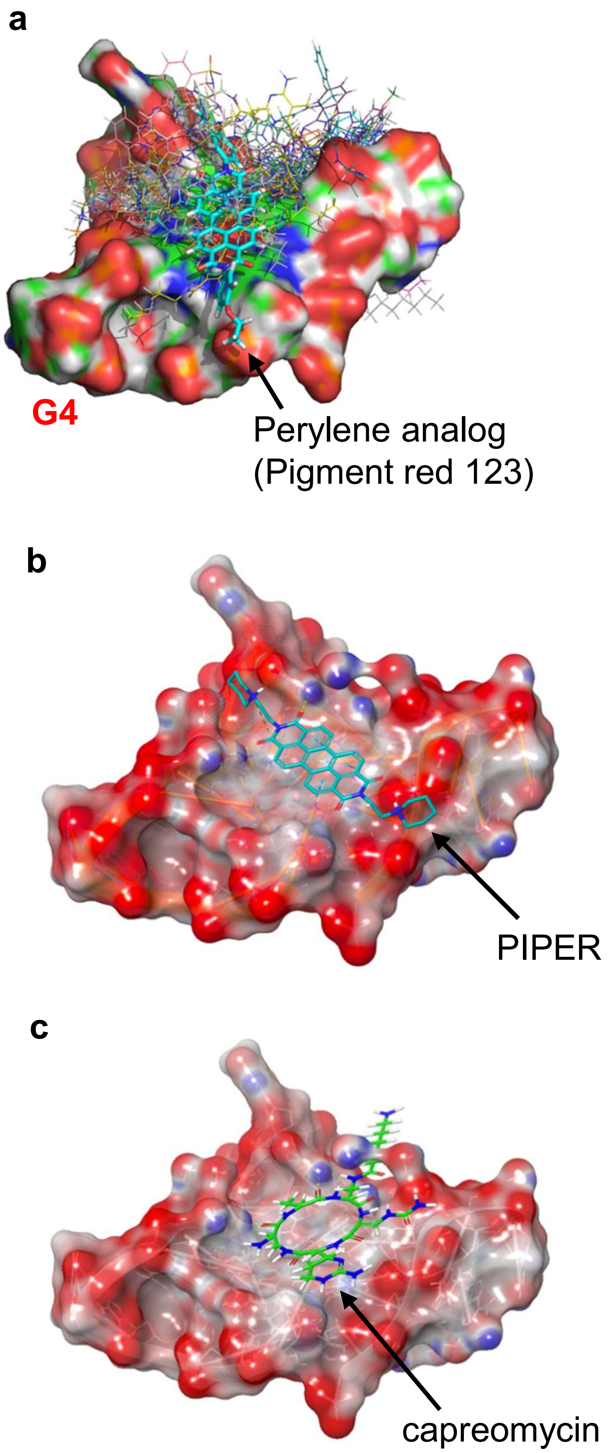

Supplementary Fig. 7 | Virtual screening to identify potential ECCs that bind to G4. a Docking different ECCs onto a G4 structure (PDB 3uyh). Docking of a known G4 binding compound, perylene analog Pigment Red 123 is specified; **b, c** Modeling of the known G4 binding ECC PIPER (**b**) and a candidate G4-binding ECC, capreomycin (**c**), onto the G4 structure. The aromatic region of the PIPER molecule (light blue) (**b**) and the capreomycin molecule (green) (**c**) extend diagonally across the planar surface of the tetrad. The side chains of PIPER are embedded in the grooves (**b**), while the capreomycin molecule shows slight favoring towards the 5' end of the G4 (**c**). The electron density surface for the 3uyh structure is shown and depicts the regions of most positive potential (dark blue) and most negative potential (dark red).

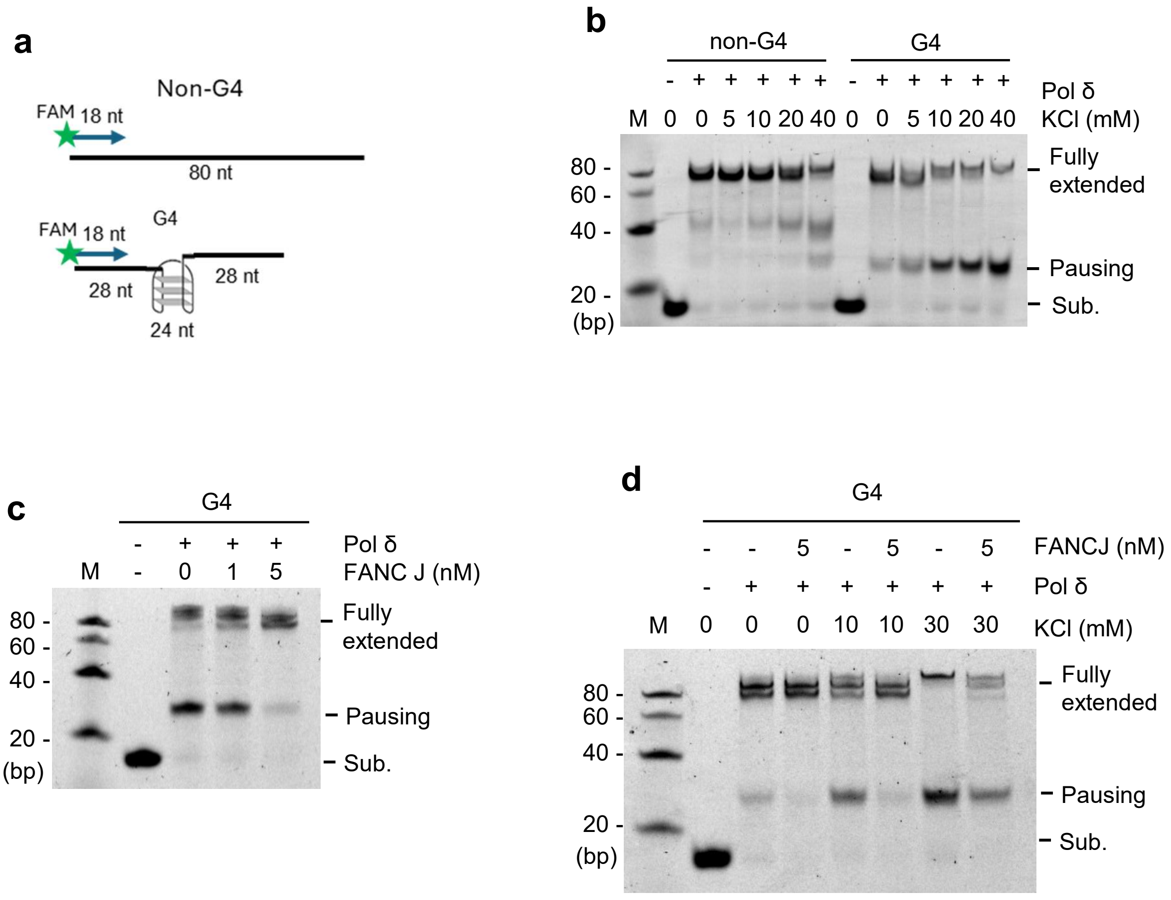

Supplementary Fig. 8 | Primer extension on the non-G4 and G4 DNA template. a The diagram shows the Polδ-catalyzed primer extension on the template without G4 (non-G4) or with G4 (G4) forming sequence. The primer was labeled with FAM on the 5' end; **b** Polδ-catalyzed primer extension on the template of non-G4 and G4 in the presence of higher concentration of KCl; **c** Polδ-catalyzed primer extension on the template of G4 with different concentrations of FANCJ; **d** Polδ-catalyzed primer extension on the template of G4 with FANCJ in the presence of different concentrations of KCl. Created in BioRender. Zhou, T. (2025) https://BioRender.com/13xuvn3

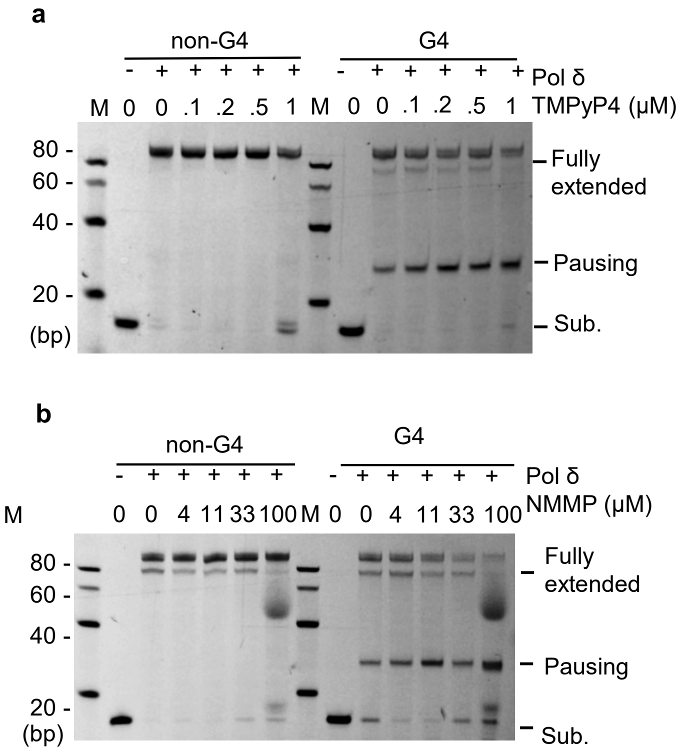

Supplementary Fig. 9 | Impact of G4-stabilizing compounds TMPyP4 and NMMP on primer extension on the non-G4 and G4 DNA template. a, b Polδ-catalyzed primer extension on the template of non-G4 and G4 in the presence of increasing concentrations of TMPyP4 (**a**) and NMMP (**b**).

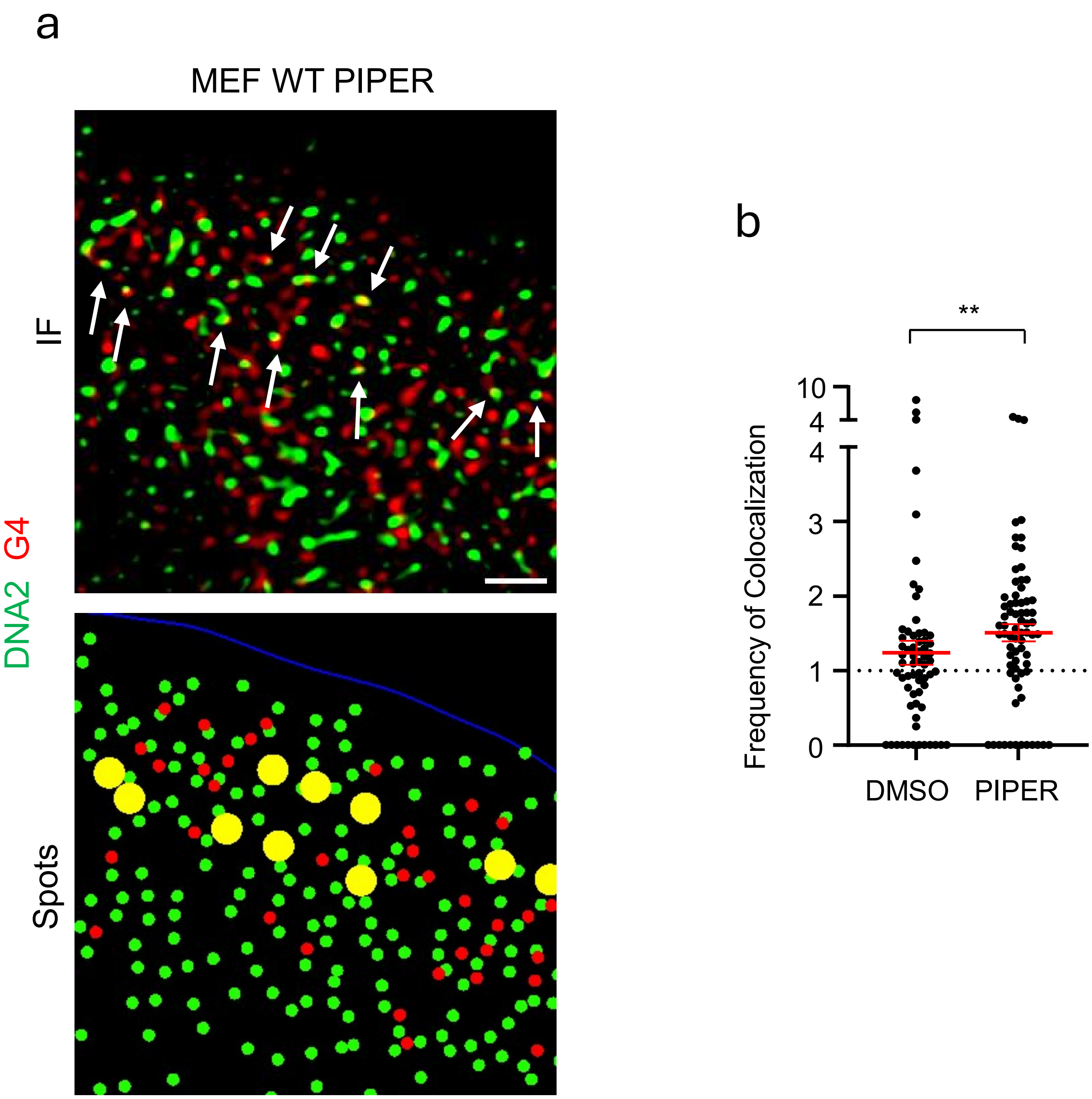

Supplementary Figure 10. G4 stabilizers cause an increased association of DNA2 at G4 regions. WT MEFs were treated with PIPER and then fixed and stained for both DNA2 and G4 (a). The relative association of DNA2 with G4 was increased in the WT PIPER condition (b). (n=64, 72 cells). P-value was calculated using two-tailed Mann-Whitney test (p=0.0028).
