# Supplementary material for: DNA2 and MSH2 cooperatively repair stabilized G4 and allow efficient telomere replication
Source: Nat Commun. 2025 Sep 26;16:8519. doi: 10.1038/s41467-025-63505-z (PMC12474859; doi:10.1038/s41467-025-63505-z)
Supplement: Supplementary file 2 — Reporting Summary [file 41467_2025_63505_MOESM2_ESM.pdf]

nature portfolio

Corresponding author(s): Binghui Shen, Li Zheng

Last updated by author(s): Jul 31, 2025

Reporting Summary

Nature Portfolio wishes to improve the reproducibility of the work that we publish. This form provides structure for consistency and transparency in reporting. For further information on Nature Portfolio policies, see our Editorial Policies and the Editorial Policy Checklist.

Statistics

For all statistical analyses, confirm that the following items are present in the figure legend, table legend, main text, or Methods section.

n/a	Confirmed	
☐	☒	The exact sample size (*n*) for each experimental group/condition, given as a discrete number and unit of measurement
☐	☒	A statement on whether measurements were taken from distinct samples or whether the same sample was measured repeatedly
☐	☒	The statistical test(s) used AND whether they are one- or two-sided<br>*Only common tests should be described solely by name; describe more complex techniques in the Methods section.*
☒	☐	A description of all covariates tested
☐	☒	A description of any assumptions or corrections, such as tests of normality and adjustment for multiple comparisons
☐	☒	A full description of the statistical parameters including central tendency (e.g. means) or other basic estimates (e.g. regression coefficient) AND variation (e.g. standard deviation) or associated estimates of uncertainty (e.g. confidence intervals)
☐	☒	For null hypothesis testing, the test statistic (e.g. *F*, *t*, *r*) with confidence intervals, effect sizes, degrees of freedom and *P* value noted<br>*Give P values as exact values whenever suitable.*
☒	☐	For Bayesian analysis, information on the choice of priors and Markov chain Monte Carlo settings
☒	☐	For hierarchical and complex designs, identification of the appropriate level for tests and full reporting of outcomes
☒	☐	Estimates of effect sizes (e.g. Cohen's *d*, Pearson's *r*), indicating how they were calculated

Our web collection on statistics for biologists contains articles on many of the points above.

Software and code

Policy information about availability of computer code

Data collection	Mass spectrometry data were collected with Proteome Discoverer Software with Sequest (Version 2.0) and the Mascot algorithm (Mascot 2.5.1). Imaging was performed with Zen Blue versions 3.6 and 3.8. Modeling of G4 with ligands was performed on Schrödinger Maestro (Version 13.9.138, MMshare Version 6.5.138, Release 2024-1, Platform Darwin-x86_64).
Data analysis	GraphPad Prism (10.4.0), ImageJ (1.54f), Adobe Illustrator (29.6.1)

For manuscripts utilizing custom algorithms or software that are central to the research but not yet described in published literature, software must be made available to editors and reviewers. We strongly encourage code deposition in a community repository (e.g. GitHub). See the Nature Portfolio guidelines for submitting code & software for further information.

Data

Policy information about availability of data

All manuscripts must include a data availability statement. This statement should provide the following information, where applicable:

- Accession codes, unique identifiers, or web links for publicly available datasets
- A description of any restrictions on data availability
- For clinical datasets or third party data, please ensure that the statement adheres to our policy

"The mass spectrometry proteomics data have been deposited to the ProteomeXchange Consortium via the PRIDE [1] partner repository with the dataset identifier PXD059843 and 10.6019/PXD059843".

Research involving human participants, their data, or biological material

Policy information about studies with human participants or human data. See also policy information about sex, gender (identity/presentation), and sexual orientation and race, ethnicity and racism.

Reporting on sex and gender	No human participants were in this study.
Reporting on race, ethnicity, or other socially relevant groupings	No human participants were in this study.
Population characteristics	No human participants were in this study.
Recruitment	No human participants were in this study.
Ethics oversight	No human participants were in this study.

Note that full information on the approval of the study protocol must also be provided in the manuscript.

Field-specific reporting

Please select the one below that is the best fit for your research. If you are not sure, read the appropriate sections before making your selection.

☒ Life sciences ☐ Behavioural & social sciences ☐ Ecological, evolutionary & environmental sciences

For a reference copy of the document with all sections, see nature.com/documents/nr-reporting-summary-flat.pdf

Life sciences study design

All studies must disclose on these points even when the disclosure is negative.

Sample size	No statistical method was used to predetermine the sample size. Sample size was determined to be adequate based on convergence and effect.
Data exclusions	No data excluded
Replication	At least three independent replicates were performed for all experiments.
Randomization	Experiments were based on cells and DNA molecules chosen at random.
Blinding	Blinding was not performed as data were collected and analyzed by the same researcher.

Reporting for specific materials, systems and methods

We require information from authors about some types of materials, experimental systems and methods used in many studies. Here, indicate whether each material, system or method listed is relevant to your study. If you are not sure if a list item applies to your research, read the appropriate section before selecting a response.

Materials & experimental systems

n/a	Involved in the study	
☐	☒	Antibodies
☐	☒	Eukaryotic cell lines
☒	☐	Palaeontology and archaeology
☐	☒	Animals and other organisms
☒	☐	Clinical data
☒	☐	Dual use research of concern
☒	☐	Plants

Methods

n/a	Involved in the study	
☒	☐	ChIP-seq
☒	☐	Flow cytometry
☒	☐	MRI-based neuroimaging

Antibodies

Antibodies used	The following antibodies were used:<br><br>Millipore Sigma

Mouse monoclonal Anti-DNA G-quadruplex (G4) Antibody (MABE1126) (4119707)

ThermoFisher Scientific
DNA2 Polyclonal Antibody (PA5-115131)
Goat anti-Mouse IgG (H+L) Highly Cross-Adsorbed Secondary Antibody, Alexa Fluor™ 568 (A11031)
Goat anti-Rat IgG (H+L) Cross-Adsorbed Secondary Antibody, DyLight™ 488 (SA5-10018) (ZF4358691)
Goat anti-Rabbit IgG (H+L) Cross-Adsorbed Secondary Antibody, Alexa Fluor™ 647 (A-21244)

BD Biosciences
BD™ Purified Mouse Anti-BrdU (347580) (3016583)
MSH6 Antibody (610918) (7319785)

Abcam
Anti-DNA2 Antibody (ab96488) (1014291-4)
Anti-BrdU antibody [BU1/75 (ICR1)] - Proliferation Marker (ab6326) (1009715-6)

Vector Laboratories
Goat Anti-Avidin D Antibody, Biotinylated (BA-0300-.5) (ZH1229)

Cell Signaling Technology
MSH2 XP Rabbit mAb (D24B5) (2017)
DYKDDDDK Tag Mouse mAb (9A3) (8146)
GAPDH Rabbit mAb (14C10) (2118)
Goat anti-rabbit HRP secondary antibody (7074)

Santa Cruz
Donkey anti-goat IgG-HRP secondary antibody (sc-2020)
Goat anti-human MSH3 (sc-5686)

Sigma-Aldrich
Anti-FLAG M2 Magnetic Beads (M8823)

Validation

Validation statements for all antibodies listed above can be found through the following links.

Millipore Sigma
https://www.emdmillipore.com/US/en/product/Anti-DNA-G-quadruplex-G4-Antibody-clone-1H6,MM_NF-MABE1126

ThermoFisher Scientific
https://www.thermofisher.com/antibody/product/DNA2-Antibody-Polyclonal/PA5-115131
https://www.thermofisher.com/antibody/product/Goat-anti-Mouse-IgG-H-L-Highly-Cross-Adsorbed-Secondary-Antibody-Polyclonal/A-11031
https://www.thermofisher.com/antibody/product/Goat-anti-Rat-IgG-H-L-Cross-Adsorbed-Secondary-Antibody-Polyclonal/SA5-10018
https://www.thermofisher.com/antibody/product/Goat-anti-Rabbit-IgG-H-L-Cross-Adsorbed-Secondary-Antibody-Polyclonal/A-21244

BD Biosciences
https://www.bdbiosciences.com/en-us/products/reagents/flow-cytometry-reagents/clinical-discovery-research/single-color-antibodies-ruo-gmp/purified-mouse-anti-brdu.347580?tab=product_details
https://www.bdbiosciences.com/en-us/products/reagents/microscopy-imaging-reagents/immunofluorescence-reagents/purified-mouse-anti-msh6.610918?tab=product_details

Abcam
https://www.abcam.com/en-us/products/primary-antibodies/dna2-antibody-ab96488?srsltid=AfmBOor8DnhAL6iZEaOc-gsB-NIiJf2h2NuGQOey6w34689nUsgdKsOe
https://www.abcam.com/en-us/products/primary-antibodies/brdu-antibody-bu1-75-icr1-proliferation-marker-ab6326?srsltid=AfmBOord2CkS0C8DLkLTXAYnjo_PUbdHmz1H2p4Vfbws04MvgxQSi6yD

Vector Laboratories
https://vectorlabs.com/products/biotinylated-anti-avidin?srsltid=AfmBOoqScwGN_soO2fHyuhGVbzjkoBKBNb10Ec0I2TgADAP0P5yc6jam

Cell Signaling Technology
https://www.cellsignal.com/products/primary-antibodies/msh2-d24b5-xp-rabbit-mab/2017?srsltid=AfmBOordMuCB47t2DzM3uszeRXY3_qs66fybm2iS0uskXFRa3jIOsJYf
https://www.cellsignal.com/products/primary-antibodies/dykddddk-tag-9a3-mouse-mab-binds-to-same-epitope-as-sigma-aldrich-anti-flag-m2-antibody/8146
https://www.cellsignal.com/products/primary-antibodies/gapdh-14c10-rabbit-mab/2118
https://www.cellsignal.com/products/secondary-antibodies/anti-rabbit-igg-hrp-linked-antibody/7074

Santa Cruz
https://www.scbt.com/p/donkey-anti-goat-igg-hrp?srsltid=AfmBOoo6VxPbPPKoW48ywUeKpgXsjfQ7er5VAkAhxIN9m936py3082zE

https://datasheets.scbt.com/sc-5686.pdf

Sigma-Aldrich
https://www.sigmaaldrich.com/US/en/product/sigma/m8823?utm_source=google&utm_medium=cpc&utm_campaign=8890193254&utm_content=87903050565&gad_source=1&gbraid=0AAAAAD8kLQT7OdU22XFgB2LaNcGkhZXBF&gclid=Cj0KCQiA-aK8BhCDARIsAL_-H9lpMgtIXWedgnsDjc3Xd07SSYBTPPuojEkVaQ6_Fq4xkAu_JKQ59mQaAinjEALw_wcB

Eukaryotic cell lines

Policy information about cell lines and Sex and Gender in Research

Cell line source(s)	whild type, DNA2+/-, MSH2-/-, and MSH6-/- Mouse Embryonic Fibroblasts (MEF cells); HEK293T cells
Authentication	MEF cells were isolated from mouse embryos in our laboratory and genotyped for DNA2. The genotypes of DNA2+/– MEF cells were confirmed by PCR analysis in our laboratory.<br>MSH2-/- and MSH6-/- MEF cells were provided by Dr. Winfried Edelmann and the genotypes confirmed by PCR analysis.<br>HEK293T cells were obtained from ATCC (American Type Culture Collection).The identity of HEK293T cells was authenticated by STR profiling performed by ATCC before shipment.
Mycoplasma contamination	All cell lines were regularly tested for mycoplasma contamination using PCR-based detection and found to be negative.
Commonly misidentified lines (See ICLAC register)	None of the cell lines used in this study were listed as commonly misidentified lines according to the ICLAC register.

Animals and other research organisms

Policy information about studies involving animals; ARRIVE guidelines recommended for reporting animal research, and Sex and Gender in Research

Laboratory animals	The study did not involve laboratory animals.
Wild animals	The study did not involve wild animals.
Reporting on sex	The study did not involve animals.
Field-collected samples	The study did not involve samples collected from the field.
Ethics oversight	The study required no ethical approval because no studies were conducted on animals.

Note that full information on the approval of the study protocol must also be provided in the manuscript.

Plants

Seed stocks	The study did not use plants.
Novel plant genotypes	The study did not use plants.
Authentication	The study did not use plants.
